# Nucleocytoplasmic human O-GlcNAc transferase is sufficient for O-GlcNAcylation of mitochondrial proteins

**DOI:** 10.1042/BCJ20160092

**Published:** 2016-06-10

**Authors:** Riccardo Trapannone, Daniel Mariappa, Andrew T. Ferenbach, Daan M.F. vanAalten

**Affiliations:** * School of Life Sciences, University of Dundee, Dundee DD1 4HN, U.K.; † Division of Molecular Microbiology, College of Life Sciences, University of Dundee, Dundee DD1 4HN, U.K.

**Keywords:** alternative splicing, glycobiology, mitochondria, O-GlcNAc transferase (OGT), O-linked *N*-acetylglucosamine (O-GlcNAc), post-translational modification (PTM)

## Abstract

O-linked *N*-acetylglucosamine modification (O-GlcNAcylation) is a nutrient-dependent protein post-translational modification (PTM), dynamically and reversibly driven by two enzymes: O-GlcNAc transferase (OGT) and O-GlcNAcase (OGA) that catalyse the addition and the removal of the O-GlcNAc moieties to/from serine and threonine residues of target proteins respectively. Increasing evidence suggests involvement of O-GlcNAcylation in many biological processes, including transcription, signalling, neuronal development and mitochondrial function. The presence of a mitochondrial O-GlcNAc proteome and a mitochondrial OGT (mOGT) isoform has been reported. We explored the presence of mOGT in human cell lines and mouse tissues. Surprisingly, analysis of genomic sequences indicates that this isoform cannot be expressed in most of the species analysed, except some primates. In addition, we were not able to detect endogenous mOGT in a range of human cell lines. Knockdown experiments and Western blot analysis of all the predicted OGT isoforms suggested the expression of only a single OGT isoform. In agreement with this, we demonstrate that overexpression of the nucleocytoplasmic OGT (ncOGT) isoform leads to increased O-GlcNAcylation of mitochondrial proteins, suggesting that ncOGT is necessary and sufficient for the generation of the O-GlcNAc mitochondrial proteome.

## INTRODUCTION

O-linked *N*-acetylglucosamine modification (O-GlcNAcylation) is a dynamic post-translational modification (PTM), characterized by the reversible covalent attachment of *N*-acetylglucosamine on serine and threonine residues of target proteins [1–3]. Over the last decade, its primary role in many biological processes in eukaryotes has been established [4–8] although many of the mechanisms underlying O-linked *N*-acetylglucosamine (O-GlcNAc) function remain to be elucidated. Interestingly, many O-GlcNAc proteins are also phosphorylated and evidence of a degree of interplay between phosphorylation and O-GlcNAcylation has been reported [7,9,10]. O-GlcNAc cycling is driven by the concerted activity of two enzymes, O-GlcNAc transferase (OGT) and O-GlcNAcase (OGA) that catalyse the attachment and the removal of O-GlcNAc respectively [11–13]. Although substrate specificity and regulatory mechanisms of OGT and OGA remain largely elusive, they are essential for stem cell viability and mouse embryonic development [14,15]. In addition, impaired O-GlcNAc metabolism has been implicated in several human diseases, such as diabetes mellitus and neurodegenerative disorders [7,8,16–18]. O-GlcNAcylation is a nutrient-dependent process and appears to be regulated by intracellular glucose availability [19,20]. Approximately 2–5% of the intracellular glucose is metabolized through the hexosamine biosynthetic pathway, a metabolic process that utilizes one of the metabolites from glycolysis to produce UDP-GlcNAc, which is the high energy donor substrate of OGT [11,21]. Human OGT (hOGT) is a 116 kDa enzyme containing a C-terminal catalytic domain and 12 N-terminal tetratricopeptide repeats (TPRs) that are known to be involved in protein–protein interactions [22]. A single *hOgt* gene has been mapped to the X chromosome at Xq13.1, a region associated with Parkinson's disease [23]. Four transcripts have been reported to originate from the *Ogt* gene in human, rat and mouse [24,25], possibly by alternative splicing or alternative promoters. Western blots performed on HeLa cell lysates, using affinity-purified rabbit polyclonal OGT antibodies, showed the presence of two distinct bands. The 116 kDa band corresponds to the nucleocytoplasmic OGT (ncOGT). An additional band, migrating at approximately 103 kDa, has been proposed to be a different splicing isoform [25]. Interestingly, the longest of the putative *Ogt* transcripts (approximately 9.5 kb) is characterized by a very long 5′-UTR, originating from the retention of intron 4 (also referred to as exon 5). This long RNA has been detected in several human and mouse tissues by Northern blot. This region, in the human gene, contains a predicted translation start site that, if utilized, would code for an OGT lacking the first three TPRs but possessing a unique 50 residue N-terminus, arising from the retained intron. This putative protein, containing nine TPRs and the catalytic domain, should result in a 103 kDa isoform [25]. Prediction tools identify a non-canonical mitochondrial targeting sequence (MTS) within the unique N-terminal region of such protein [26]. This 103 kDa band is enriched in the mitochondrial fraction in HeLa cells, and it has been referred to as mitochondrial OGT (mOGT) [26]. This proposed isoform has been shown to be active *in vitro* [27], whereas its overexpression leads to apoptosis in some cell lines [28]. Catalytically inactive GFP-tagged mOGT, overexpressed in HeLa cells, is targeted to mitochondria, whereas in the absence of the predicted MTS, it is localized in the cytosol [26]. The biological role of mOGT in human cell lines has not been investigated. It has been suggested to be involved in O-GlcNAcylation of mitochondrial proteins, apoptosis and/or metabolic pathways [29]. Functional consequences of nuclear and cytoplasmic O-GlcNAcylation have been extensively studied, whereas very little is known about the mechanistic biology resulting from mitochondrial protein O-GlcNAcylation. Initially, no O-GlcNAc was detected in mitochondrial fractions by Western blot [26]. Nevertheless proteomics and more sensitive anti-O-GlcNAc antibodies have overcome this issue. Notably, mitochondrial O-GlcNAcylated proteins have been identified in mouse cardiac myocytes, rat heart and rat liver by mass spectrometry (MS) [30–33]. Moreover, the presence of OGT and OGA has been detected by immunogold labelling in mitochondria [34]. Interestingly, increased mitochondrial protein O-GlcNAcylation, due to hyperglycaemic conditions in cardiac myocytes, has been associated with modulation of the electron transport chain activity, oxygen consumption rate, ATP production and calcium uptake [30,35]. Similarly, 2D electrophoresis experiments have shown that mitochondrial protein O-GlcNAc modification and phosphorylation patterns are altered in myoblasts exposed to high glucose concentrations [36]. More recently, Tan et al. [37] have demonstrated that overexpression of ncOGT or OGA alters protein expression levels in mitochondria and severely affects mitochondrial morphology and metabolic processes. O-GlcNAcylation of dynamin-related protein 1 (Drp1), which is one of the main regulators of mitochondrial dynamics, induces mitochondrial fragmentation and altered membrane potential in cardiac myocytes [38]. Another O-GlcNAcylated protein is trafficking kinesin-binding protein 1 (TRAK1)/Milton, which is known to form a stable complex with ncOGT and with the kinesin mitochondrial transport machinery [39,40]. Increased O-GlcNAcylation of TRAK1 in hyperglycaemic conditions leads to altered mitochondrial axonal transport [41]. Finally, increased O-GlcNAcylation in aging rat retina has been proposed to have a protective effect on mitochondrial respiration and dynamics as well as redox homoeostasis, helping prevent reactive oxygen species (ROS)-related aging [42]. Together, these studies suggest a potential and largely unexplored link between O-GlcNAc cycling and many key biological functions carried out by mitochondria, such as ATP production, lipid metabolism, apoptosis and ROS homoeostasis, although it is not clear whether O-GlcNAc directly regulates these processes [43]. Exploration of the role of O-GlcNAc in mitochondrial physiology may uncover links with mitochondrial dysfunction, dynamics and transport in neurodegenerative, neuroinflammatory and autoimmune diseases [44,45].

In this work, we probed the presence and role of the previously reported mOGT isoform in cell lines and animal tissues. We also studied the contribution of ncOGT and mOGT in generating the mitochondrial O-GlcNAc proteome. Surprisingly, it appears that ncOGT is sufficient for O-GlcNAcylation of mitochondrial proteins, in agreement with mOGT being undetectable.

## MATERIALS AND METHODS

### Cloning and primers

The previously reported mOGT coding region [26] was amplified by PCR (SuperScript III One-step RT PCR kit, Invitrogen) from human cDNA using the following sets of primers: 5′-aaaggatccatgctgcagggtcacttttggctgg-3′ and 5′-cgtc-tcaattgctttcaaataacatgcc-3′ first, followed by 5′-cgagatccattgt-gctggatctacaatgctgcagggtcacttttggc-3′ and 5′-cgtctcaattgctttcaa-ataacatgcc-3′. The resulting PCR product was cloned in a pEBG6P backbone according to published methods [46] to generate a tagless construct. To add a C-terminal FLAG-tag, the QuikChange site-directed mutagenesis kit (Stratagene) was used with the following primers: 5′-gcctg-ttgaagtcactgagtcagcagctgactacaaggacgatgacgataagtaagcggccgcg-actctagagtgag-3′ and 5′-ctcactctagagtcgcggccgcttacttatcgtcatcgt-ccttgtagtcagctgctgactcagtgacttcaacaggc-3′.

### Cell culture and transfection

Human embryonic kidney (HEK) 293 and HeLa cells were cultured in Dulbecco's modified Eagle medium (DMEM) medium (Gibco), supplemented with 10% FBS, L-glutamine, penicillin and streptomycin, at 37°C in a humidified 5% CO_2_ incubator. HEK 293 suspension cells were grown in Pro 293s-CDM medium (Lonza), supplemented with 1% FBS, L-glutamine, penicillin and streptomycin, at 37°C in a humidified 5% CO_2_ incubator with gentle shaking. HEK 293 cells were plated on 10 cm dishes at 50% confluence and then transfected for 48 h with 10 μg of plasmid using 5 μg of polyethylenimine (PEI, Polyscience) transfection reagent. HeLa cells were plated on 10 cm dishes at 90% confluence and then transfected for 24 h with 10 μg of plasmid and 5 μl of Lipofectamine 2000 (Invitrogen) according to the manufacturer's instructions. For HEK 293 suspension cell transfection, 2×10^8^ cells were grown for 4 h in 50 ml serum-free medium in 250 ml flasks. A transfection mixture, containing 100 μg of DNA and 50 μg of PEI in 10 ml serum-free medium was added to the cells and incubated at 37°C for 4 h. Finally 1% FBS in 40 ml medium was added to the cells up to a final volume of 100 ml and cells were incubated with gentle shaking at 37°C for 72 h. For knockdown experiments, HeLa cells were plated on six-well plates at 70% confluence and transfected for 48 h and 96 h with 100 pmol ON-TARGETplus OGT set of four siRNA (GE Dharmacon) using 2 μl of Lipofectamine 2000. After 48 h, growth medium was changed and fresh transfection mixture was added to increase silencing efficiency. For HeLa cell mOGT transfection, in combination with siRNA, cells were transfected at the same time with 100 pmol ON-TARGETplus OGT set of four siRNA and 10 μg of mOGT construct, in the presence of 5 μl Lipofectamine 2000. Cells were then incubated for 48 h.

### RNA extraction and PCR

Mouse tissues were obtained from adult female mice after cervical dislocation. Total RNA was isolated from tissue and from cultured human cell lines using the TRI-Reagent (Sigma–Aldrich) according to the manufacturer's instructions. Ten micrograms of each RNA sample was digested with DNAse (New England BioLabs) to eliminate genomic DNA contamination. cDNA was prepared with the Precision nanoScript reverse transcription kit (Primer Design), using 1 μg of starting material. The following sets of primers were used to amplify different regions of the *Ogt* gene in human and mouse samples (Figure 2): (1) forward 5′-ctcaaagccctgggtcgctt-3′, reverse 5′-gcaaagttcggttgcgtctc-3′; (2) forward 5′-gccatacctc-ttaacacctc-3′, reverse 5′-gcaaagttcggttgcgtctc-3′; (3) forward 5′-ccctgggtcgcttggaagaa-3′, reverse 5′-ggatgaaattcttctccact-3′; (4) forward 5′-ttctgatgatgcgctgtgac-3′, reverse 5′-aggctcgcttg-cgccaaa-3′. PCR products were analysed on 1% agarose gel containing GelRed fluorescent staining (Biotium).

### Purification of mitochondria and protease protection assay

Mitochondria were purified from mouse liver and HEK 293 suspension cells as described previously [47] with minor modifications. Unless specified, all steps were carried out at 4°C. Briefly, 4×10^8^ cells were washed in ice-cold PBS, pH 7.5, resuspended in 10 ml of mitochondria isolation buffer (MIB: 10 mM Tris/MOPS, pH 7.4, 1 mM EGTA/Tris, 200 mM sucrose) and disrupted with a Glass-Teflon homogenizer (Thomas Scientific, 20–25 passes). Mouse liver was quickly extracted from female mice after cervical dislocation, washed four times in 50 ml of ice-cold MIB and minced in small pieces with scissors. The tissue was then resuspended in 10 ml of MIB and homogenized (25–30 passes). Cell or liver homogenates were spun at 600 ***g*** for 10 min. Pellets, containing nuclei and unbroken cells, were collected for SDS/PAGE, whereas supernatants were spun again at 600 ***g*** for 10 min to remove cell debris and remaining nuclei. Supernatants were transferred into 30 ml round-bottomed glass centrifuge tubes and spun at 7000 ***g*** for 10 min. After centrifugation, supernatants, mainly containing the cytosolic fraction, were collected for further analysis. Pellets, mainly containing the mitochondrial fraction, were resuspended in 10 ml of MIB and spun again at 7000 ***g*** for 10 min to remove cytosolic contaminants. Mitochondria-enriched pellets were then collected and used for subsequent experiments. Protease protection experiments were performed as described elsewhere [48,49]. Briefly, freshly isolated mitochondria were resuspended in MIB containing 10 μg/ml proteinase K (Sigma–Aldrich) and incubated on ice for 30 min with gentle mixing every 5 min. Proteolysis was terminated by adding 5 mM PMSF and Laemmli sample buffer and by heating the samples at 95°C for 10 min.

### Cell lysis and immunoprecipitation

Cell or tissue samples were washed in ice-cold PBS, homogenized in a glass homogenizer where necessary, and incubated for 10–30 min in lysis buffer (50 mM Tris/HCl, 1 mM EGTA, 1 mM EDTA, 1% Triton X100, 1 mM sodium orthovanadate, 50 mM NaF, 5 mM sodium pyrophosphate, 0.27 M sucrose, 0.1% 2-mercaptoethanol and Complete™ protease inhibitor cocktail containing 1 mM benzamidine, 0.2 mM PMSF and 5 μM leupeptin) on ice. Lysates were spun at 15000 ***g*** for 10 min at 4°C. Supernatants were collected for immunoprecipitation experiments or diluted in Laemmli sample buffer containing 5% 2-mercaptoethanol for SDS/PAGE. Immunoprecipitation experiments were carried out by incubating cell/tissue lysates (between 0.5–4 mg) with OGT antibody (DM-17, Sigma–Aldrich) at 4°C with gentle rolling overnight. Subsequently, protein-G Dynabeads (Life Technologies) were added to the lysate for 2 h at 4°C with gentle rolling. Dynabeads were then washed thrice with 1 ml of washing buffer (20 mM Tris/HCl, pH 7.4, 150 mM NaCl, 1 mM EDTA, 0.05% Triton X100, 5% glycerol and Complete™ protease inhibitor cocktail). Finally, beads were incubated with Laemmli sample buffer containing 5% 2-mercaptoethanol and heated at 95°C for 10 min to elute proteins.

### SDS/PAGE and Western blot analysis

For Western blot analysis, protein samples were separated by SDS/PAGE and transferred on to nitrocellulose membrane (Whatman). The membranes were blocked for 1 h in 5% non-fat milk powder or 5% BSA in TBS-0.2% Tween20 (TBS-T) buffer for 1 h at room temperature with gentle shaking. Then, the following primary antibodies, diluted in blocking buffer, were added to the membranes overnight at 4°C: anti-OGT (DM-17, Sigma–Aldrich), anti-OGT (Abcam ab177941), anti-O-GlcNAc (RL2, Abcam), anti-O-GlcNAc [C-terminal domain (CTD) 110.6, Cell Signaling], anti-TOM20 (F-10, Santa Cruz Biotechnology), anti-ATP synthase β subunit (ATPB, 3D5, Abcam), anti-Timm13 (A01, Abnova), anti-HSP60 (D307, Cell Signaling), anti-α-tubulin (12G10, Developmental Studies Hybridoma Bank, University of Iowa) and anti-α-tubulin (11H10, Cell Signaling). The following fluorescent secondary antibodies, diluted in 1% milk in TBS-T, were added to the membranes for 1 h at room temperature: donkey anti-rabbit 800, donkey anti-mouse 680, goat anti-rabbit 800 and goat anti-mouse 680 (LI-COR). Images were acquired and analysed using the LI-COR Odyssey Image System.

## RESULTS

### The mOGT ORF is not conserved in mammals

**Figure 1:**
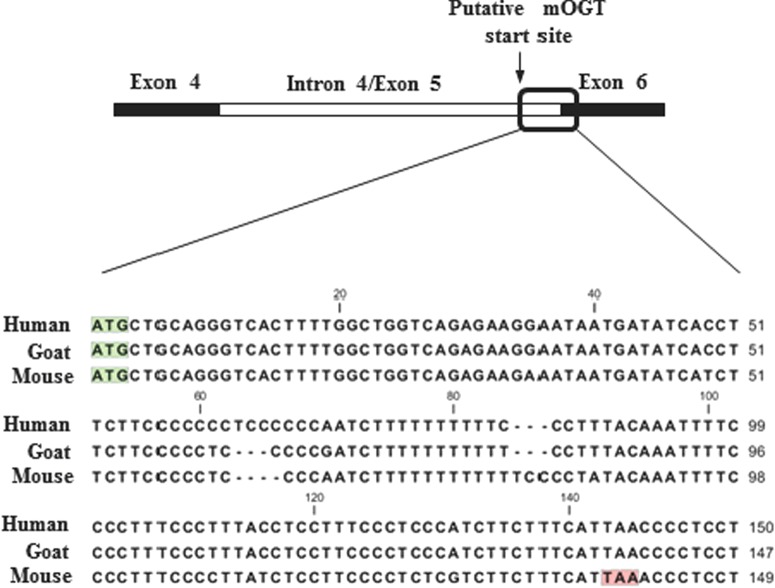
Conservation of mOGT The 150 bp region between the predicted mOGT start site within intron 4/exon 5 and exon 6 in the *hOgt* gene was aligned with the homologous region in other species. Example of alignment between the human, goat and mouse sequences is shown. The green boxes indicate the putative mOGT start codon. The red box in the mouse sequence highlights the stop codon.

**Figure 2:**
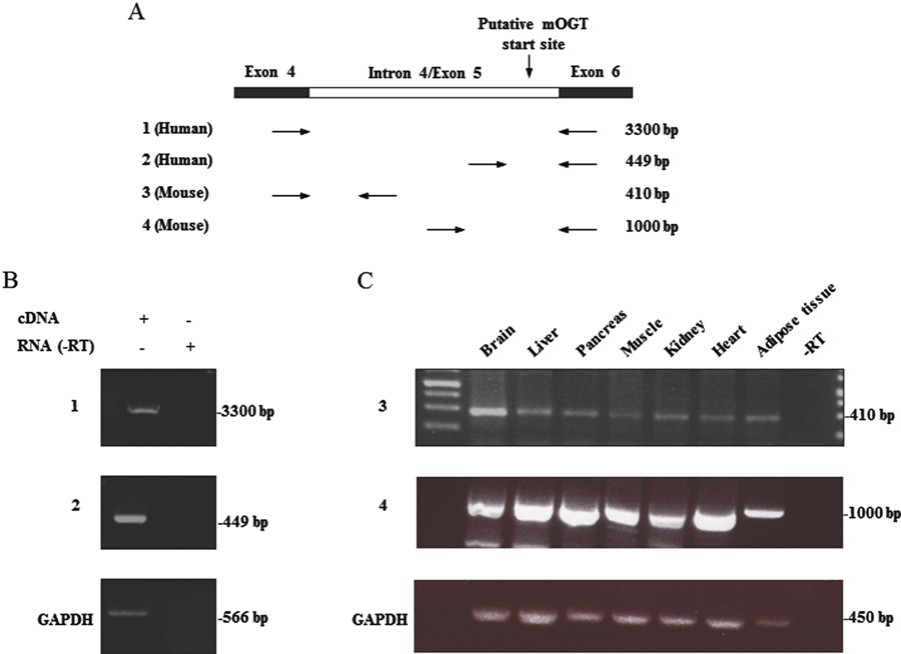
Analysis of mOGT-coding transcripts in human cell lines and mouse tissues (**A**) PCR was performed on cDNA prepared from human HEK 293 cells and mouse tissues. Different primer combinations, specific for the human and the murine *Ogt* sequence, allowed detection of the retained intronic sequence. Sizes of expected PCR products are also indicated. Amplified DNA samples from human (**B**) and mouse (**C**) were analysed on agarose fluorescent gel. An mRNA sample not subjected to reverse transcriptase PCR (-RT) is included as a negative control. Glyceraldehyde 3-phosphate dehydrogenase (GAPDH) was used as a normalizing control.

The conservation and presence of mOGT across the mammalian kingdom has not been studied. To predict the presence of mOGT protein, sequence alignment of the genomic region coding for the putative human mOGT ORF and the *Ogt* gene in other species was performed. The mOGT translation start site of the human gene lies within intron 4 (also referred to as exon 5), 150 bp from the intron/exon boundary (Figure 1). This region shows high homology among all the species analysed (Table 1). Surprisingly, when the homologous sequences were analysed using the ExPAsy translate tool to predict ORFs, a shift in the reading frame was observed, leading to the introduction of a premature stop codon, in mouse as well as most of the other species analysed (Figure 1 and Table 1). This appears to exclude the possibility of a functional mOGT protein being translated from an ORF through a mechanism similar to that in human. Only some primates, cow and goat contain a putative ORF, although the presence of mOGT in these organisms has never been investigated at the protein level. We also analysed sequence similarity in lower organisms including *Drosophila*, *Caenorhabditis elegans* and zebrafish, but in these cases no significant homology of intron 4 was detected. The reading frame shift was confirmed in mouse liver genomic DNA by sequencing PCR products. Hence, a more detailed investigation on the retention of intron 4/exon 5 in human and mouse was carried out. For this purpose, reverse transcriptase PCRs to detect the transcript carrying intron 4/exon 5 were performed on mRNA from HEK 293 cells and mouse tissues, using four primer combinations (Figure 2A). Results from this experiment demonstrate that the transcript carrying intron 4/exon 5 is present both in mouse and human (Figures 2B and 2C).

**Table 1:** Bioinformatic analysis of predicted presence of mOGT ORFs

Species	Sequence identity	Gaps	mOGT ORF
*Gorilla gorilla* (gorilla)	100%	0/150	Yes
*Pongo pygmaeus* (orangutan)	100%	0/150	Yes
*Macaca mulatta* (macaque)	99%	1/150	No
*Callithrix jacchus (*marmoset*)*	97%	4/150	No
*Capra hircus* (goat)	95%	3/150	Yes
*Sus scrofa* (pig)	95%	5/150	No
*Canis familiaris* (dog)	95%	5/150	No
*Ovis aries* (sheep)	95%	5/150	No
*Felis catus* (cat)	94%	3/150	No
*Equus caballus* (horse)	94%	6/150	No
*Bos taurus* (cow)	93%	9/150	Yes
*Oryctolagus cuniculus* (rabbit)	93%	3/150	No
*Rattus norvegicus* (rat)	90%	6/150	No

### Endogenous mOGT protein is not detectable

**Figure 3:**
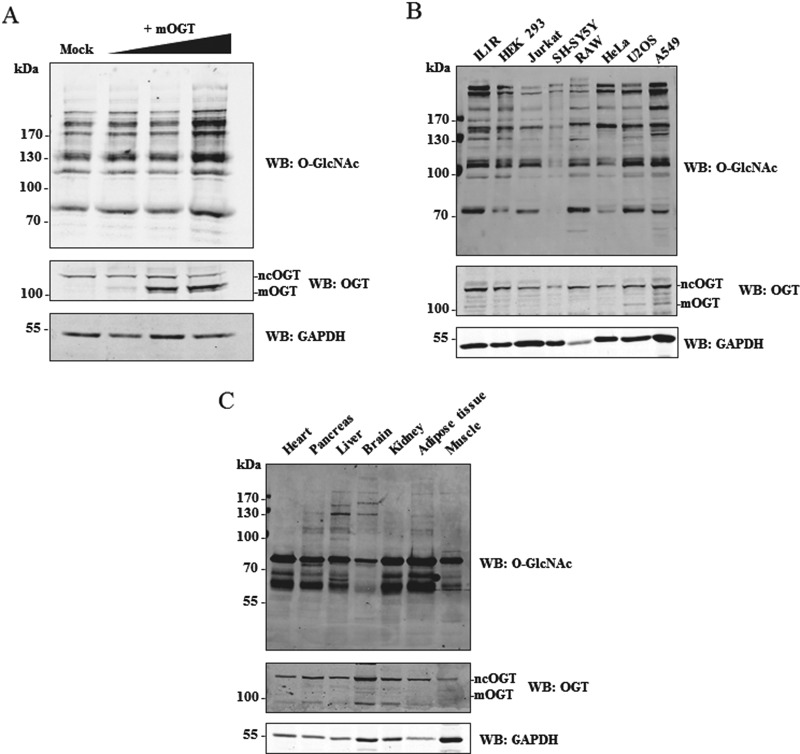
Detection of endogenous mOGT (**A**) Varying amounts (1–5 μg) of a tagless mOGT construct were overexpressed in HEK 293 cells and lysates were probed with anti-O-GlcNAc (RL2), anti-OGT (DM-17) and anti-GAPDH antibodies. (**B**) Expression of endogenous mOGT was assessed in different cell lines: HEK 293 suspension IL1R, HEK 293, Jurkat, SH-SY5Y, RAW, HeLa, U2OS and A549. Lysates were probed with anti-O-GlcNAc (RL2), anti-OGT (DM-17) and anti-GAPDH antibodies. (**C**) Mouse tissue lysates were probed with the same antibodies.

**Figure 4:**
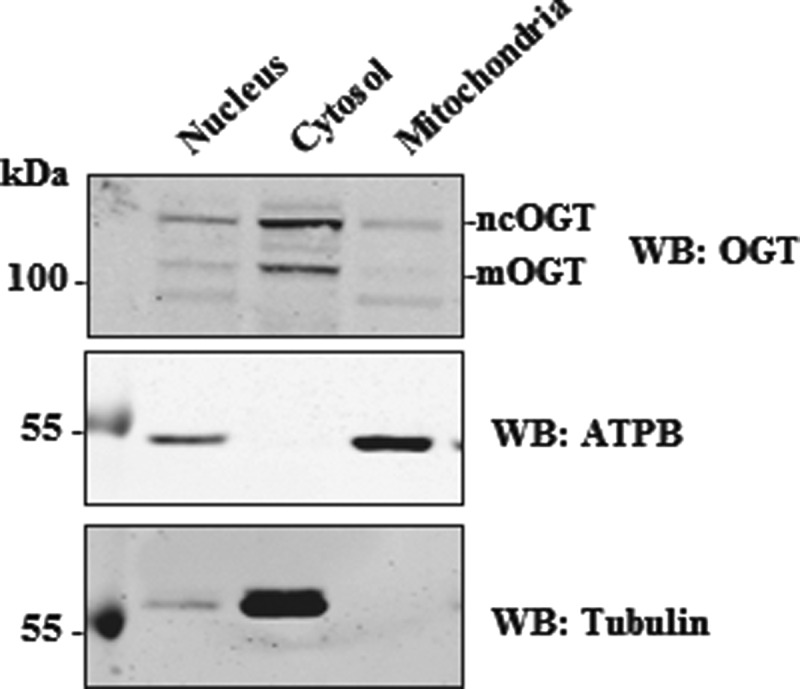
Putative mOGT is not enriched in mitochondrial fractions Subcellular fractionation was performed in HEK 293 cells: nuclear, cytosolic and mitochondrial fractions were analysed by Western blot.

mOGT was previously identified by Western blot in HeLa cells, although its presence in other human cell lines or tissues has not been explored [26]. We probed lysates from several cultured human cell lines with a commercial OGT antibody (Sigma clone DM-17) to assess mOGT expression levels. This antibody was raised against a synthetic peptide corresponding to amino acids 740–756 of the C-terminus of ncOGT and it should detect all of the predicted OGT isoforms. In agreement with published data, ncOGT can be clearly detected on Western blot, with varying expression levels in different cell lines and conditions (Figure 3). To confirm that the DM-17 anti-OGT antibody can indeed detect mOGT, lysates from HEK 293 cells, transfected with a tagless mOGT construct, were analysed by Western blot (Figure 3A). Results show that the DM-17 antibody is able to detect overexpressed mOGT. An additional band, at a molecular mass (103 kDa) compatible with mOGT, was also detected (Figure 3B). Lysates from different mouse tissues were also probed with DM-17 antibody (Figure 3C). The 103 kDa mOGT band is not detected in the mouse tissues analysed, but several non-specific bands appeared with high exposures. An attempt at generating an mOGT-specific antibody using the predicted unique N-terminus as antigen was unsuccessful. Localization of the putative mOGT in human cell lines was investigated. Total cell and mitochondrial lysates from HEK 293 cells were subjected to OGT DM-17 Western blot analysis (Figure 4). Two distinct bands can be observed in total lysates, supposedly corresponding to ncOGT and mOGT, but neither of these two bands is distinctly enriched in the mitochondrial fraction. Although weak bands are detectable in mitochondria, this could be due to contamination from other cell compartments.

**Figure 5:**
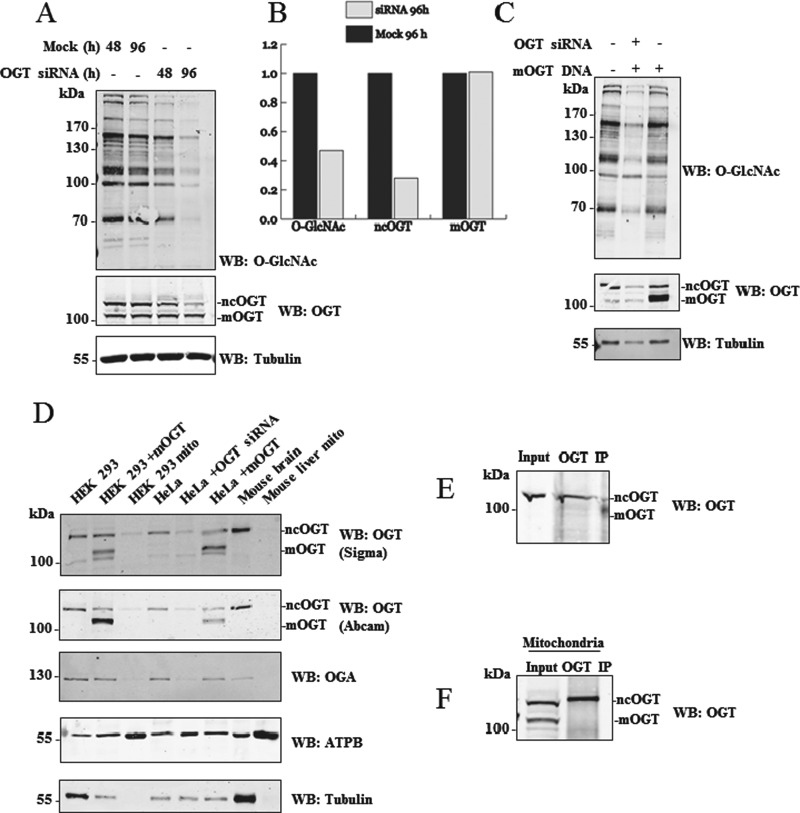
The 103 kDa band is not mOGT (**A**) OGT siRNA was performed in HeLa cells for 48 h and 96 h. Lysates were analysed by Western blot. (**B**) Densitometry analysis of the blots shown in (A). (**C**) A combination of siRNA and mOGT overexpression was also used to test the specificity of the siRNA. (**D**) Different lysates (HEK 293 total lysate, HEK 293 with mOGT overexpression, HEK 293 mitochondrial fraction, HeLa total lysate, HeLa with OGT siRNA, HeLa with mOGT overexpression, mouse brain total lysate, mouse liver mitochondrial fraction) were probed with two different OGT commercial antibodies (DM-17 Sigma and ab177941 Abcam). ATPB and α-tubulin served as mitochondrial and cytoplasmic marker respectively. OGT was immunoprecipitated from different untransfected HEK 293 lysates: total cell lysate (**E**) or HEK 293 mitochondrial fraction (**F**). Anti-OGT antibody (DM-17) was used for immunoprecipitation.

To ascertain the specificity of the putative 103 kDa mOGT band, knockdown of OGT was attempted using OGT siRNA. A combination of four siRNAs covering the whole *Ogt* gene sequence was used in HeLa cells for 48 h and 96 h. These should theoretically knockdown any predicted OGT isoform. Western blot analysis showed a strong reduction in global O-GlcNAc levels, in particular with 96 h siRNA transfection (Figures 5A and 5B). Similarly, ncOGT expression was reduced by approximately 70% upon knockdown. Surprisingly, the 103 kDa putative mOGT band was not affected by the treatment (Figures 5A and 5B). The ability of the siRNA to knockdown the putative mOGT transcript was tested by overexpressing mOGT. Overexpressed mOGT protein levels were significantly reduced upon OGT siRNA treatment, demonstrating that the mOGT transcript is susceptible to knockdown with these reagents (Figure 5C). These data suggest that the endogenous putative mOGT immunogen may be a non-specific band detected by the DM-17 OGT antibody. To further dissect this, another commercial OGT antibody (Abcam), also raised against a portion of the catalytic domain (between amino acid 1000 and the C-terminus of ncOGT), was used. Both antibodies are able to detect ncOGT both in human and in mouse tissue total lysates but no specific band is detected in mitochondrial fractions (Figure 5D). A relatively faint 103 kDa band, detected by the DM-17 antibody in total lysates, is not reactive to the Abcam antibody, even at higher exposure. Given that both antibodies can detect overexpressed mOGT, this suggests that the 103 kDa band may not correspond to mOGT. Endogenous ncOGT but not the 103 kDa band, could be immunoprecipitated from total HEK 293 lysate or the mitochondrial fraction with the DM-17 antibody (Figures 5E and 5F). This precluded identification of the 103 kDa band by MS or N-terminal sequencing.

### ncOGT is sufficient for O-GlcNAcylation of mitochondrial proteins

**Figure 6:**
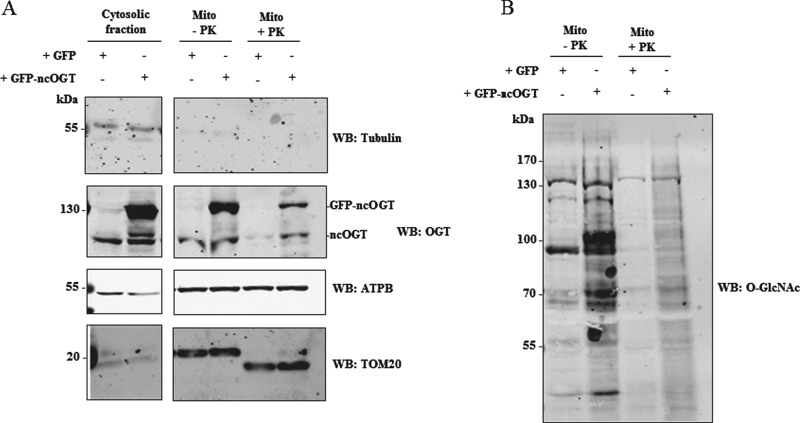
ncOGT is sufficient for O-GlcNAcylation of mitochondrial proteins (**A**) HEK 293 suspension cells were transfected with either GFP or GFP-tagged ncOGT. Mitochondria, purified from transfected cells, were incubated with buffer or with 10 μg/ml of proteinase K. Lysates from the mitochondrial or the cytosolic fractions were probed with anti-OGT, anti-α-tubulin (cytosol), anti-ATPB (mitochondrial matrix) and anti-TOM20 (mitochondrial outer membrane) antibodies. (**B**) O-GlcNAc levels in mitochondrial fractions were detected with anti-O-GlcNAc antibody (CTD 110.6). The amount of protein loaded in each lane in (A) is 10 μg, whereas approximately 100 μg was used in (B).

Given the apparent presence of only a single detectable OGT isoform, it is worth exploring whether this isoform is sufficient for generating the mitochondrial O-GlcNAc proteome. GFP-tagged ncOGT was overexpressed in HEK 293 suspension cells. Mitochondria were purified from cells and incubated with either buffer or buffer containing proteinase K to digest the outer membrane-associated proteins and contaminants. Overexpression of ncOGT leads to increased O-GlcNAc in the mitochondrial fraction (Figure 6B) and the same increase is visible in the protease-treated sample, demonstrating that overexpressed ncOGT is able to transfer O-GlcNAc on to mitochondrial proteins. Interestingly, a trace of ncOGT is still visible in the mitochondrial fraction after protease treatment, suggesting that part of ncOGT itself might be imported into mitochondria and be involved in O-GlcNAc cycling inside the organelle.

## DISCUSSION

After the first report describing mOGT in 2003 [26], there have been no further reports on this isoform for more than a decade, whereas numerous studies have begun to uncover the functions of ncOGT. Recent and previous proteomics data have revealed the presence of O-GlcNAc modified mitochondrial proteins [30,32,35]. Moreover, accumulating evidence suggests that O-GlcNAcylation is an important regulator of many biological processes related to mitochondrial function [8]. For instance, O-GlcNAc appears to be important for mitochondrial function and dynamics, by mechanisms not clearly understood [30,34,37]. However, it is still unclear whether and how much O-GlcNAc directly affects mitochondrial metabolism [43]. The possible functional link between O-GlcNAc and mitochondrial function served as a starting point of this work, where we planned to explore the role of mOGT in human and mouse and to investigate how mOGT generates the mitochondrial O-GlcNAc proteome. However, sequence alignments of intron 4/exon 5 in the predicted hOGT ORF with the homologous region in other species revealed the presence of a stop codon in most of the species analysed, except some primates, goat and cow (Figure 1 and Table 1). Human and mouse transcripts, retaining the putative mOGT N-terminus, were detected (Figure 2). Data shown in this work are in agreement with previous Northern blot experiments [24,25] showing the existence of several transcripts derived from the *Ogt* gene in various human and mouse samples. The presence of many transcripts was also documented in zebrafish [50], where different splice variants are differentially expressed at mRNA level throughout development. Intron retention is a quite common event and long untranslated RNAs were reported elsewhere [51]. Therefore, it is possible that some of the *Ogt* transcripts observed have functions other than protein coding. The genomic alignments shown in this work suggest that there is no mOGT ORF in the mouse, precluding it as an animal model to study the function of mOGT. The lack of mOGT in most species might suggest that its function is not essential or that there is redundancy between ncOGT and mOGT. The next step was to investigate the presence and biological role of mOGT in human cell lines. This isoform was previously detected using a non-commercial (and unavailable) rabbit affinity-purified antibody [25,26]. We have made several attempts to identify mOGT in human cell lysates and mitochondrial fractions. Only one of the two commercial antibodies tested was able to detect a band at the right molecular mass, although results were inconsistent and variable (Figure 3). Overexpressed mOGT, however, was readily detected by either antibody used (Figures 3A and 5D). To further determine the specificity of the antibodies, siRNA was performed in HeLa cells, using a combination of four RNAs specific for both ncOGT and mOGT. Results showed that only ncOGT protein levels were reduced, whereas the putative mOGT band, as well as other non-specific bands, was not affected (Figures 5A and 5B). The presence of additional non-specific bands was previously reported using in-house developed rabbit antisera [11] or other commercially available anti-OGT antibodies [52]. These data suggest that the putative mOGT band detected with DM-17 antibody might be non-specific. The same band is not enriched in HEK 293 mitochondrial fraction (Figure 4). In summary, we could not detect mOGT protein under the experimental conditions we used. It cannot be excluded that this particular OGT isoform is expressed only transiently under very specific conditions that are yet to be established.

Given the apparent absence of mOGT, we then explored whether ncOGT itself may produce the mitochondrial O-GlcNAc proteome. We showed that overexpressed GFP-tagged ncOGT is able to increase O-GlcNAc on mitochondrial proteins (Figure 6). Protease treatment of mitochondria from transfected cells excludes the possibility that the increase in the O-GlcNAc levels we observe is due to contaminants or outer membrane-associated proteins. The idea of ncOGT being solely responsible for O-GlcNAcylation of mitochondrial proteins is also supported by the presence of mitochondrial O-GlcNAcylated proteins in mouse and rat, where the mOGT protein is not present.

On the basis of the data presented here, we propose two possible models. In the first, ncOGT and OGA respectively add and remove O-GlcNAc from mitochondrial proteins in the cytosol prior to import into mitochondria. In the second, the two O-GlcNAc cycling enzymes are imported into the organelle and perform their catalytic activity within mitochondria. Most of the mitochondrial proteome is encoded by the nuclear genome, translated in the cytosol and then transported into mitochondria. This observation may support the first scenario. Nevertheless, Figure 6(A) shows the presence of residual ncOGT in mitochondrial fractions upon protease treatment, suggesting that a portion of ncOGT is imported into the organelle. Recent immunogold labelling experiments have shown the presence of OGT and OGA in mitochondria [34]. Interestingly, the pyrimidine nucleotide carrier 1 (pnc1) has been shown to be responsible for transport of UDP-GlcNAc into mitochondria [34]. Knockdown of this transporter led to decreased mitochondrial O-GlcNAcylation [34], suggesting the possibility of O-GlcNAc cycling occurring within the organelle. This second scenario is supported by immunoprecipitation experiments detecting O-GlcNAc on cytochrome c oxydase I (COX I) [30] and a proteomic screen mapping an O-GlcNAc site on NADH-ubiquinone oxidoreductase chain 4 (mt-ND4) [35]. Both proteins are encoded by the mitochondrial DNA and belong to the electron transport chain. Therefore, they are never exposed to the cytoplasm and would thus need to be modified in the mitochondrial matrix. It is possible that both models are valid and different mitochondrial substrates are reversibly O-GlcNAcylated either in the cytosol or in the matrix/inner membrane. Other PTMs have been reported in mitochondria, including phosphorylation and acetylation [53], although it is not clear whether the modification takes place in the cytosol before protein import or if the enzymes responsible for these processes are also present inside mitochondria.

Taken together, the data shown in this work suggest that ncOGT is the main isoform responsible for the generation of the mitochondrial O-GlcNAc proteome. However, it is not possible to exclude the presence of additional OGT isoforms encoded by a different gene or another type of mitochondrial OGT. Further studies are necessary to identify O-GlcNAcylated mitochondrial proteins in human and to investigate the biological role of O-GlcNAc on specific proteins in the context of mitochondrial dysfunction and disease.

## Abbreviations

ATPB: ATP synthase β subunit
COX I: cytochrome c oxydase I
CTD: C-terminal domain
DMEM: Dulbecco's modified Eagle medium
Drp1: dynamin-related protein 1
GAPDH: glyceraldehyde 3-phosphate dehydrogenase
HEK: human embryonic kidney
hOGT: human OGT
MIB: mitochondria isolation buffer
mt-ND4: NADH-ubiquinone oxidoreductase chain 4
MTS: mitochondrial targeting sequence
mOGT: mitochondrial OGT
ncOGT: nucleocytoplasmic OGT
OGA: O-GlcNAcase
O-GlcNAc: O-linked N-acetylglucosamine
O-GlcNAcylation: O-linked *N*-acetylglucosamine modification
OGT: O-GlcNAc transferase
PEI: polyethylenimine
PTM: post-translational modification
ROS: reactive oxygen species
TPR: tetratricopeptide repeat
TRAK1: trafficking kinesin-binding protein 1
